# Recycling of Heterogeneous Mixed Waste Polymers through Reactive Mixing

**DOI:** 10.3390/polym15061367

**Published:** 2023-03-09

**Authors:** Vincenzo Titone, Emmanuel Fortunato Gulino, Francesco Paolo La Mantia

**Affiliations:** 1 Department of Engineering, University of Palermo, Viale delle Scienze, 90128 Palermo, Italy; 2 INSTM, Consortium for Materials Science and Technology, Via Giusti 9, 50125 Florence, Italy

**Keywords:** heterogeneous blend, recycling, polyolefin, mechanical properties, rheology

## Abstract

Anything that is not recycled and/or recovered from waste represents a loss of raw materials. Recycling plastics can help to reduce this loss and to reduce greenhouse gases, improving the goal of the decarbonization of plastic. While the recycling of single polymers is well assessed, the recycling of mixed plastics is very difficult because of the strong incompatibility among the different polymers usually present in urban waste. In this work, heterogeneous mixed polymers, i.e., polyethylene (PE), polypropylene (PP), polystyrene (PS) and polyethylenetherephthalate (PET) were processed using a laboratory mixer under different conditions of temperature, rotational speed and time to evaluate the effect of the above parameters on morphology, viscosity and mechanical properties of the final blends. Morphological analysis shows a strong incompatibility between the polyethylene matrix and the other dispersed polymers. The blends show, of course, a brittle behavior, but this behavior slightly improves with decreasing temperature and increasing rotational speed. A brittle-ductile transition was observed only at a high level of mechanical stress obtained by increasing rotational speed and decreasing temperature and processing time. This behavior has been attributed to both a decrease in the dimensions of the particles of the dispersed phase and to the formation of a small amount of copolymers that act as adhesion promoters between matrix and dispersed phases.

## 1. Introduction

Recycling heterogeneous mixed plastics waste is a very difficult challenge because of the strong incompatibility of the different chemical and molecular structures of the polymers composing the mixture [1]. This problem is usually overcome for two phase polymer blends by using a third component that reduces the interfacial tension between the two phases improving adhesion and decreasing the dimensions of the minor components in the matrix [2]. This third components can be a copolymer partly compatible with both components or a functionalized polymer partly compatible with one of the two phases and that reacts with the second component giving rise to a copolymer compatible with both phases. This copolymer acts as a compatibilizer improving the adhesion between the two components. In this case the compatibilization is called reactive blending and many functionalized macromolecules [3,4,5,6] or degraded polymers with oxygenated groups [7] have been reported. Of course, this is not possible, or extremely difficult, in the presence of more polymers. Processing mixtures with different polymers such as polyolefins, polyesters or polyamides gives rise to a polymer system fragile with very poor mechanical properties, bad aesthetic appearance, etc. [8]. A second and not negligible problem is connected with the different melting points of the different components. Mixing at the processing temperature of the highest melting point polymer could, indeed, imply a severe thermal or thermomechanical degradation of the other components. It should be then necessary to work at the lowest possible temperature for short times. These two conditions have been successfully used in two pieces of equipment for the production of multicomponent mixtures with good final properties [9,10,11].

In the paper [8], mixtures of strongly incompatible polymers such as polyolefins, polyvinylchloride and polyethylenetherephthalate are mixed in an unconventional mixer at low temperature, even below the melting temperature of some components and at high shear stress, producing a blend with good mechanical properties. It has been demonstrated that during the mixing in these extreme processing conditions it is possible to form copolymers that act as compatibilizers among the different phases, Similar conditions are used in the so called “solid shear stress pulverization” [10,11] to obtain, in an ad-hoc designed twin screw extruder, blends with good mechanical properties starting from heterogeneous polymer mixtures.

Both processes are then based on three basic principles: (1) low temperature, even lower than the melting temperature of the highest melting temperature polymers; (2) short processing times; (3) high shear stresses [9,10,11]. The first two conditions reduce the effects of thermal degradation, while the high shear stresses can break the macromolecules producing radicals that, reacting among them, can form copolymers that can compatibilizer the mixture [12]. Of course, the limit of these processes is that they need special, ad hoc equipment.

Recently, various approaches based on the use of cryogenic temperatures [13] and reactive extrusion in the presence of compatibilizers [14,15] and nanofillers [16] are emerging to simplify the waste recycling process.

The aim of this work is to evaluate if similar results can be obtained by using conventional apparatuses such as a laboratory mixer. Moreover, the process does not consider any use of compatibilizers or, in general, any other component. A heterogeneous mixture made by polyolefins, polyethylene, polypropylene and polystyrene, and polyethylenetherephthalate has been processed in a laboratory internal mixer in different processing conditions–temperature, rotational speed and time–to evaluate the effect of time, temperature and shear stress on the morphology, on the viscosity and on the mechanical properties of the final blend.

The experimental results put in evidence that with decreasing temperature and increasing the shear stress, the morphology of the blend improves mainly because the dimensions of the dispersed phases decrease and because the adhesion seems improved due to of the formation of copolymers by reactions between the macroradicals formed because of the mechanical stress applied to the melt. In particular, the processing time plays a very important role. A fragile-to-ductile transition is observed by decreasing the mixing time. Indeed, the better blend is obtained at a low temperature and mixing time, while morphology and properties become worse with an increasing temperature and mixing time. A possible competition between the formation of copolymers that can act as compatibilizers and thermomechanical degradation of the components and of the same copolymers can interpret this behavior.

## 2. Materials and Methods

### 2.1. Materials

The main characteristics of the materials used in this work are shown in Table 1. PET comes from bottles for water. High- and low-density polyethylene (HDPE and LDPE), polyethylenetherephthalate (PET), polypropylene (PP) and polystyrene (PS) are the more used polymers for the production of rigid and flexible packaging and are the more important polymers encountered in the urban plastic waste collection.

The values for HDPE, LDPE, PP and PS were taken from data sheet [17,18,19,20], while the value of the MFI of PET was measured at 270 °C under a weight of 325 g (condition K) [21].

### 2.2. Blends Preparation

The HDPE/LDPE/PP/PET/PS mixtures were prepared according to the composition given in Table 2 by melt mixing in a Brabender mixer (Brabender, model PLE 330, Duisburg, Germany)

The blends were prepared at different temperatures (see Table 3) and rotational speeds (see Table 4).

In the processing conditions A, B and C, the crystalline fraction of PET remains in solid state. Before blending, PET was dried in a vacuum oven at 120 °C overnight.

Table 5 shows all the blend combinations investigated. For example, D2 indicates a blend processed at 270 °C (D) and 120 rpm (2), while B3 is the code of a blend processed at 210 °C (B) and 250 rpm (3).

**Figure 1:**
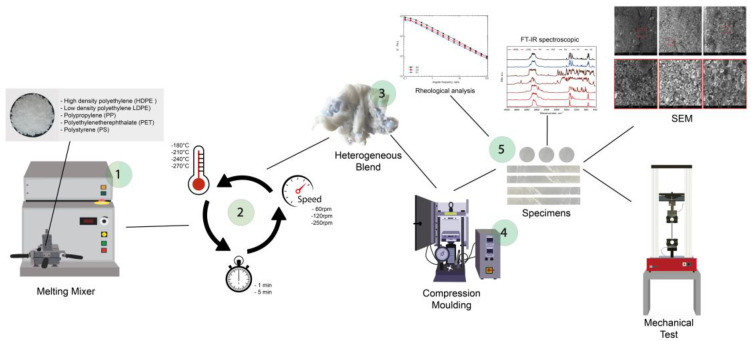
Diagram illustrating the steps in the preparation and characterization of the heterogeneous mixtures.

Figure 1 illustrates the production and characterization steps of heterogeneous mixtures.

In order to verify the presence of copolymers formed during the mixing, two binary blends were prepared: PS/PET and LDPE/PET at 180 °C, 250 rpm for 1 and 5 min. The two-blend composition was 40/60 for PS/PET and 65/35 for LDPE/PET. The same ratio between the two components is presented in the multiphase blend.

### 2.3. Characterizations

#### 2.3.1. Rheological Analysis

Melt flow index (MFI) values of all the blends were measured with a CEAST extrusion plastometer (CEAST, model. 6542, Torino, Italy) at a temperature of 270 °C under a load of 2.16 Kg.

Complex viscosity curves were obtained using an ARES G2 rotational rheometer (TA Instruments, New Castle, DE, USA). The tests were performed in parallel plates mode with a diameter of 25 mm. Shear viscosity values of all the samples were measured at 270 °C from 100 to 0.1 rad/s.

#### 2.3.2. Mechanical Analysis

Mechanical (tensile) tests were performed according to ASTM D638 -14 [22] using an Instron universal testing machine (Instron, mod. 3365, High Wycombe, U.K.). The elastic modulus was measured at the deformation rate of 1 mm/min until 3% deformation. Then, the crosshead speed was increased to 20 mm/min until the specimen failed. The reported results are an average of at least 7 measurements.

The specimens used to measure the mechanical properties were prepared by compression molding in a Carver laboratory hydraulic press (Carver, Wabash, IN, USA) at a temperature of 260 °C and a mold pressure of 300 psi for about 2 min.

#### 2.3.3. Structural and Morphological Analysis

IR spectroscopic analysis was performed to study the interactions and to analyze the specific functional groups present in the blends. Fourier transform infrared (FT-IR) spectra were performed using a Perkin-Elmer FT-IR spectrometer (Perkin-Elmer, Norwalk, CT, USA). Spectra were collected in the range 4000–400 cm^−1^ with 32 scan numbers at 4 cm^−1^.

SEM images were obtained through a Phenom proX scanning electron microscope (Phenom World, Eindhoven, Netherlands). Before examination by SEM, specimens were fractured in liquid nitrogen. Image analysis was conducted using ImageJ software, which is freely available and in the public domain.

The numerical average diameter was calculated as follows:

(1) $$D_{n}=\frac{\sum_{i}(D_{i}\times n_{i})}{\sum_{i}n_{i}}$$

The possible formation of copolymers has been monitored by dissolving the blends in a solvent of only one of the components. The presence of copolymers under the form of colloids gives some turbidity to the solutions. This test, known as the Molau test [23], has been used for blends of polyolefins and polyamides used as a solvent formic acid. In our case, the test has been used on two binary blends, PS/PET and LDPE/PET in order to verify if the polar components can form copolymers with matrix PE and with the dispensed polyolefin phase, PET. In the first case the solvent was tetrahydrofuran at room temperature, while for the LDPE the solvent was tetrahydronaphthalene at 80 °C. The suspensions obtained were analyzed visually and the turbidity was measured with a commercial portable turbidimeter (HANNA Instruments, mod. HI93102, Woonsocket, RI, USA).

## 3. Results and Discussion

### 3.1. Blends Characterization

For all the mixtures, the mixing time was 5 min, the lowest time at which all the heterogeneous mixtures reached a steady state value. This means that all the mixtures reached in these mixing conditions have a thermo-flow-dynamic equilibrium. MFI values of all the blends mixed for 5 min at all the mixing conditions are reported in Table 6.

It can be seen that the MFI value decreases as the temperature decreases and the mixing speed increases. Of course, by increasing the mixing speed, the melt is subjected to higher shear mechanical stress. For example, the D1 blend, obtained at 60 rpm, shows an MFI of 0.689 while the D3 blend, mixed at 250 rpm, shows an MFI of 0.571. A significant difference in the MFI values was also observed by changing the processing temperature. Indeed, the value of the MFI of A3, mixed at 180 °C, decreased by about 12% with respect to the blend A3 mixed at the same rotational speed, but at 270 °C.

**Figure 2:**
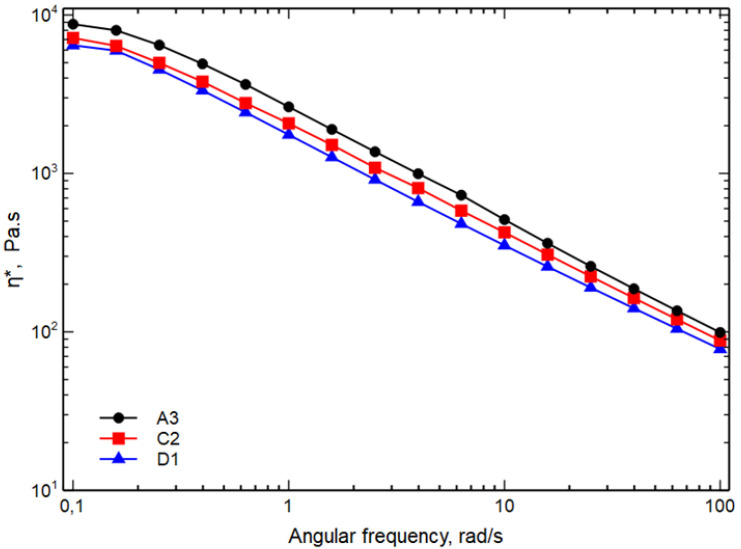
Complex viscosity as function of the frequency of the samples A3, C2 and D1.

Figure 2 shows the complex viscosity curves, η*, as a function of the frequency. For the sake of simplicity, only the viscosity curves of A3, C2 and D1 blends are shown.

As expected, from the results of MFI, it can be seen that, as the temperature decreases and the mixing speed increases, an increase in viscosity is observed both at low frequencies and at high frequencies. The viscosity of the blends depends on the viscosity of the components but also on the size of the dispersed phases and on the adhesion among the phases. In particular, the viscosity of the blends increases with decreasing the size of the particles of the dispersed phases and improving the adhesion among the phases. It is, then, possible to hypothesize that sample A3 could have dispersed phases particles with lower dimensions and/or a better adhesion between the continuous and dispersed phases.

**Figure 3:**
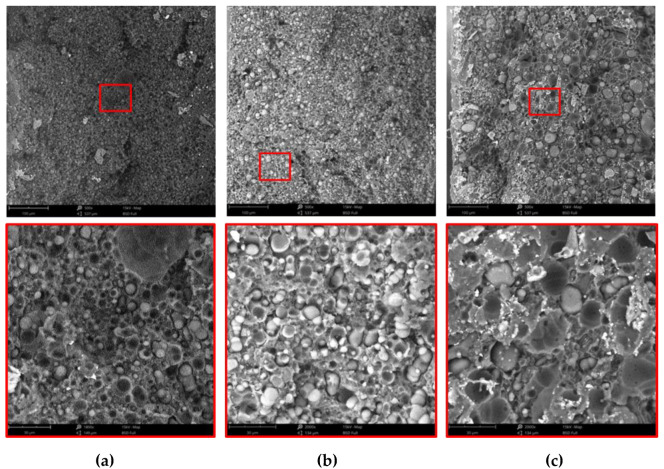
SEM micrographs: (**a**) A3, (**b**) C2 and (**c**) D1.

The micrographs of the same blends reported in Figure 2 are shown in Figure 3 at two different magnifications. Sample D1 can be considered as the reference sample because it is the only blend processed at 270 °C with all the components in a molten state similar to all the conventional melt processing operations. The micrographs clearly indicate the strong incompatibility between the polyethylene matrix and the other dispersed polymers. Indeed, this blend shows the typical morphology of heterogeneous incompatible blends. The dispersed particles are quite big, show a broad range of dimensions and many large voids are observed in the continuous phase, indicating a very scarce adhesion between matrix and dispersed phases. By decreasing the temperature and increasing the stress of samples C2 and A3, however, the size of the dispersed particles decreases suggesting a better mixing and, moreover, smaller voids seem to suggest a slightly better adhesion.

**Figure 4:**
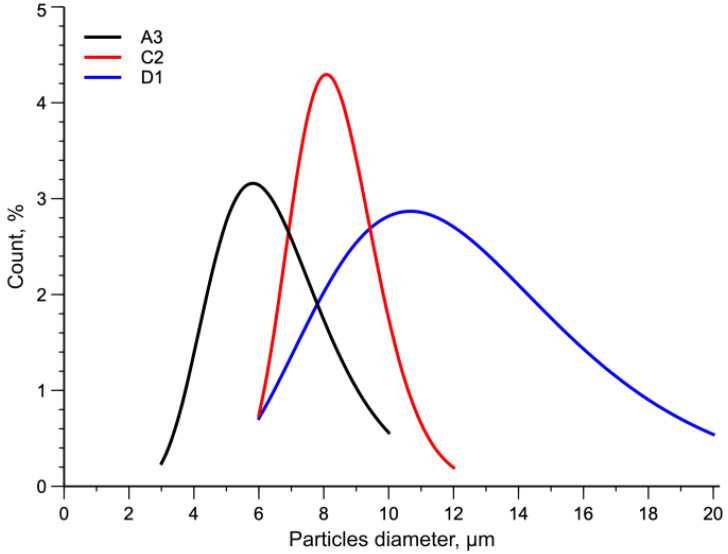
Normal distribution curves of particles diameter.

In Figure 4 the particle diameter distribution for the three samples is reported. Statistical functions were used to describe the distribution of particles. In particular, the normal distribution was used. The curves shown in the figure confirm the qualitative observations made on the micrographs.

Lower temperatures and higher rotational speeds give rise to higher shear stress acting on the melt. These processing conditions strongly determine the size of the dispersed particles and their distribution. Indeed, by decreasing the temperature from 270 °C to 180 °C and increasing the rotational speed, samples A3 and D1, the numerical average diameters are 6.5 µm and 12.6 µm, with the distribution ranges going from 3–10 µm to 6–20 µm, respectively (see Table 7).

In agreement with our previous works, [24,25] the contact area between matrix and dispersed particles was calculated by considering the surface area of each particle by the number of particles. In more detail, the contact area of each particle is proportional to its square diameter, as follows

(2) $$A_{i}\propto \;D_{i}^{2}$$

and the number of particles is as follows:

(3) $$N_{i}=\frac{V_{ti}}{V_{i}}$$

where *V_ti_* is the total volume in the sample and *V_i_* is the volume of each individual particle present. The total contact area of each sample is then as follows:

(4) $$A_{i}t=\frac{A_{i\;}}{N_{i}}=\frac{A_{i}V_{ti}}{V_{i}}\;\propto \;\frac{D_{i}^{2}V_{ti}}{D_{i}^{3}}\;\propto \;\frac{V_{ti}}{D_{i}}$$

The ratio between the contact area of A3 e C2 with respect to D1 is as follows

(5) $$A_{A3}t\;/\;A_{D1}t=\frac{V_{tA3\;}D_{D1}}{V_{tD1\;}D_{A3}}\;\;\mathrm{and}\;A_{C2}t\;/\;A_{D1}t=\frac{V_{tC2\;}D_{D1}}{V_{tD1\;}D_{C2}}$$

where $\;A_{A3}t$, $A_{C2}t$ and $A_{D1}t$ are the total contact and are of the sample A3, C2 and D1, respectively. In Table 8 the values of the total contact area with respect to D1 are reported.

Based on the average equivalent diameter reported above, it is observed that the total contact area increasing with decreasing temperature and increasing rotational speed. By increases the contact area and decreasing the particles diameter the transmission of the stress is improved giving rise to better properties of the blend.

In Table 9 the values of the torque registered after 5 min for the same samples are reported.

The torque, directly proportional to the shear mechanical stress acting on the melt, can be considered as the unique experimental processing parameter that determines the final morphology of the blend because the torque increases with increasing the mixing speed and decreasing the temperature. Higher shear stresses are able to break the particles of the dispersed phases giving rise to smaller particles and then to a decrease in the particle diameter and to an increase in the contact area between the matrix and the other phases. Moreover, it is possible to hypothesize that the very high shar stress breaks the macromolecular chains giving rise to macroradicals. The macroradicals of different polymers can react forming copolymers that can act as compatibilizers or as adhesion promoters between the matrix and other phases, improving, then the adhesion between the various polymers with the matrix.

**Figure 5:**
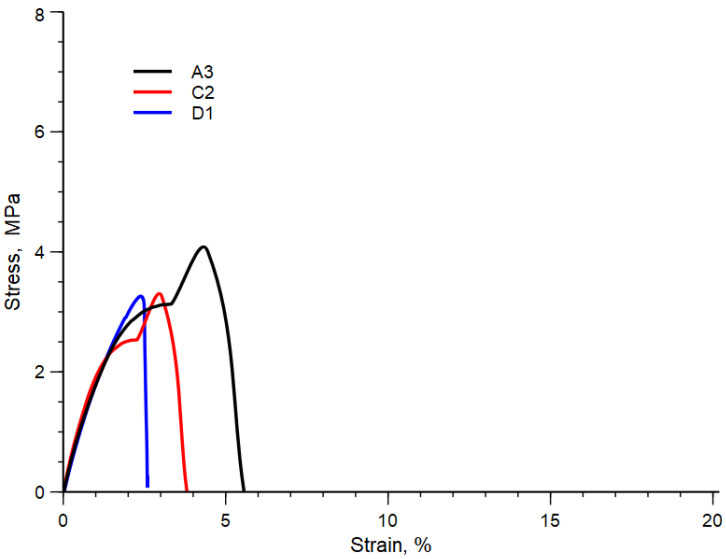
Stress-strain curve of A3, C2 and D1 blends.

Table 10 summarizes the values of elastic modulus, E, tensile strength, TS and elongation at break, EB, of all the blends analyzed, while, in Figure 5, for the sake of simplicity, the typical stress-strain curves of the blend A3, C2 and D1 are shown. In this Figure, the sudden change of the stress-strain curves is due to the change in the crosshead speed.

As expected, due to the strong heterogeneous nature of these blends, all the samples are brittle, see Figure 5, and present low mechanical properties (see Table 9). However, all the mechanical properties slightly improve with decreasing the temperature and with increasing the rotational speed. This behavior can be ascribed to both the decrease in the diameter and then to an increase in the contact area and/or to the same improvement in the adhesion due to the formation of copolymers.

The elongation at break is the mechanical property more sensible to the molecular structure and to the morphology. Then, the improved elongation at the break of the A3 blend can be attributed to the better morphology obtained at the lower temperature and higher mechanical stress. On the contrary, the worst value of elongation at the break is observed for sample D1 prepared at the highest temperature and lowest rotational speed.

**Figure 6:**
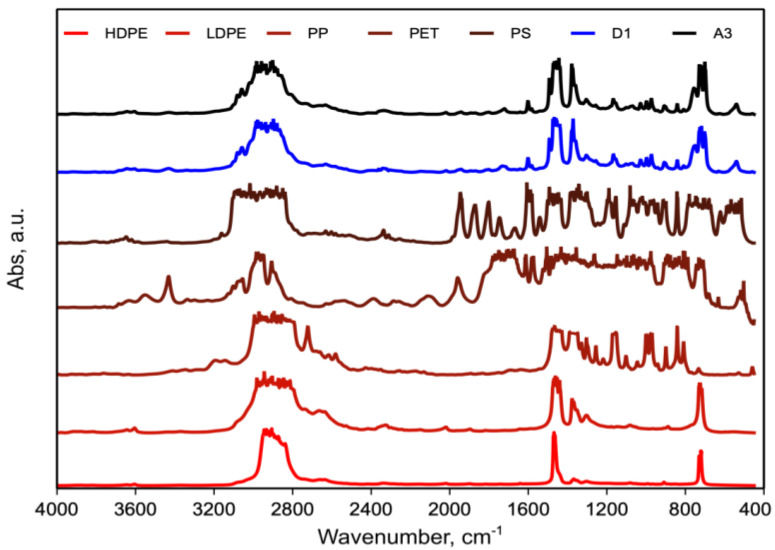
FTIR spectra of A3, D1 blend and individual polymers in blend: PE (HD and LD), PP, PET and PS.

Figure 6 shows the FTIR spectra of the A3 and D1 blends and the individual polymers in the blend.

The comparison of the FTIR spectra of pure PEs (HD and LD), PP, PET and PS with those of the blends D1 and A3 revealed that no new peaks or significant shifts of peaks were observed. However, it is not possible to ascertain if this is due to the lack of formation of copolymer or is due to instrumental limits because of the very small amount of undetectable copolymers.

### 3.2. Effect of the MIXING Time

**Figure 7:**
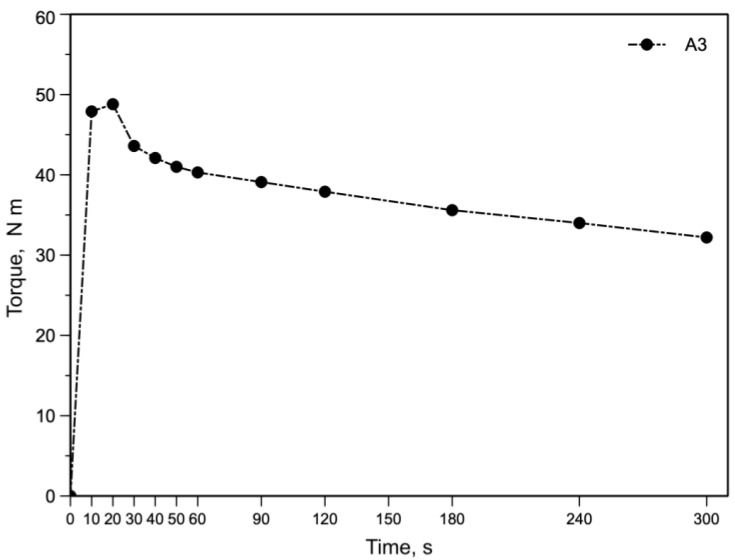
Curve of the torque as a function of mixing time for A3 sample.

All the previous results indicate that morphology, rheological and mechanical properties depend on the processing conditions and that the mechanical stress acting on the melt plays a very important role. In Figure 7 the curve of the torque as a function of the mixing time is reported for sample A3.

**Figure 8:**
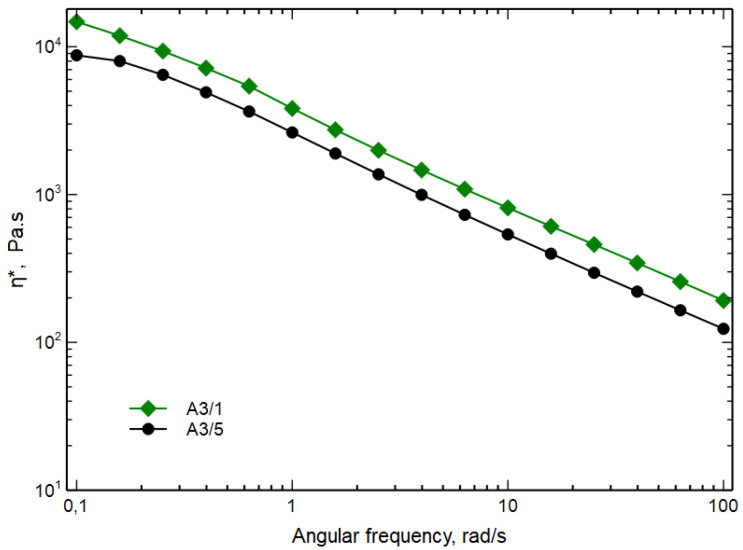
Complex viscosity as function of angular frequency of A3/1 and A3/5 blends.

Similar to all the curves of the torque in a mixing operation, the curve first increases due to the feeding of the cold polymer, reaches a maximum and then decreases reaching a thermo-flow-dynamic equilibrium suggesting that there is no more change in the morphology of the blend. Considering the previous hypothesis about the beneficial effect of high values of mechanical stress on the development of the morphology, tests have been done by stopping the mixing after 1 min, at which time a maximum is observed. The MFI values and the flow curves of the A3 samples mixed 1 min (A3/1) and 5 min (A3/5) are reported in Table 11 and in Figure 8, respectively.

The blend A3/1 shows a lower value of the MFI and a higher value of the flow curve both at low and high frequencies. In particular, sample A3/1 does not reach a Newtonian plateau in this frequency range and shows a more pronounced non-Newtonian behavior. As said before, this phenomenon is due to both the size of the dispersed particles and/or a better adhesion. From these rheological data it is, then possible to hypothesize a decrease in the dimensions of the particles and/or a better adhesion between the continuous phase and dispersed phases for sample A3/1.

**Figure 9:**
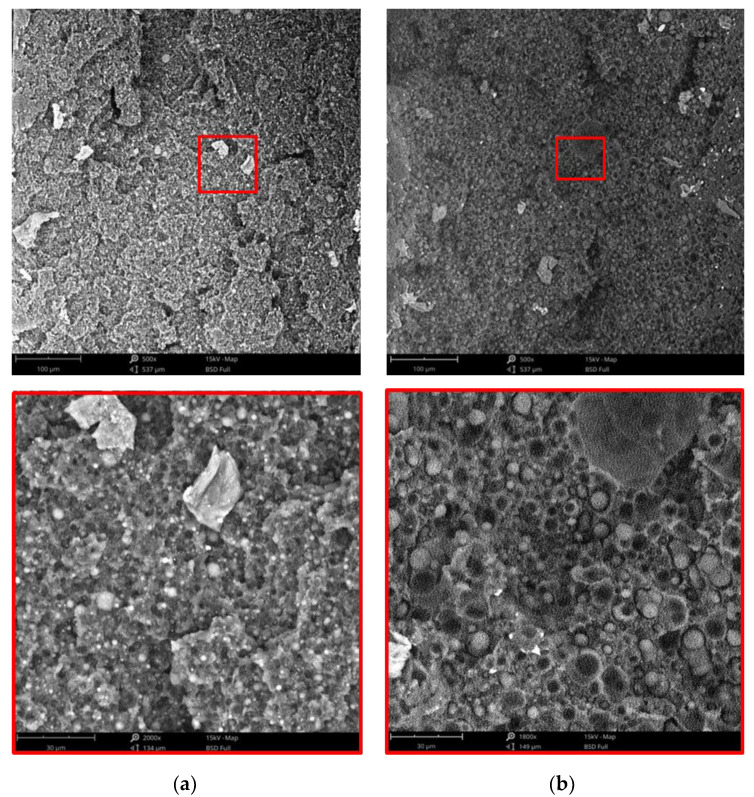
SEM micrographs: (**a**) A3/1, (**b**) A3/5 blends.

**Figure 10:**
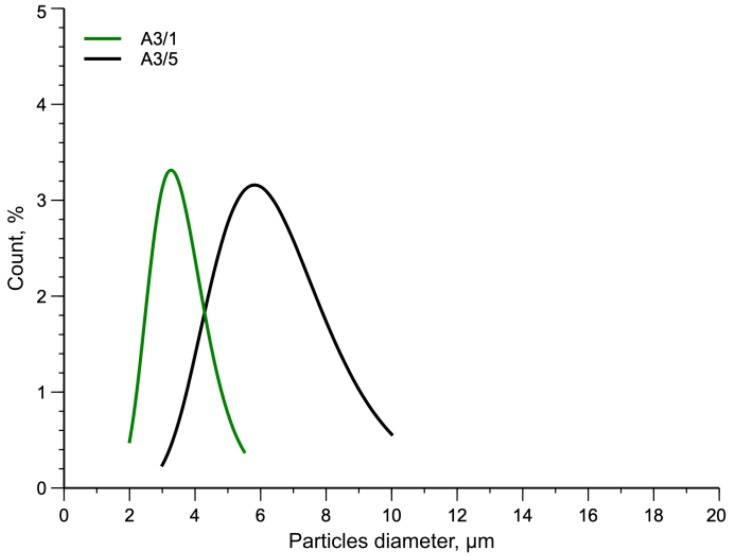
Normal distribution curves of particles diameter.

In Figure 9 the SEM micrographs of the two samples are reported. The relative curves of the particle diameter distribution and numerical average diameter values are shown in Figure 10 and Table 12, respectively.

**Figure 11:**
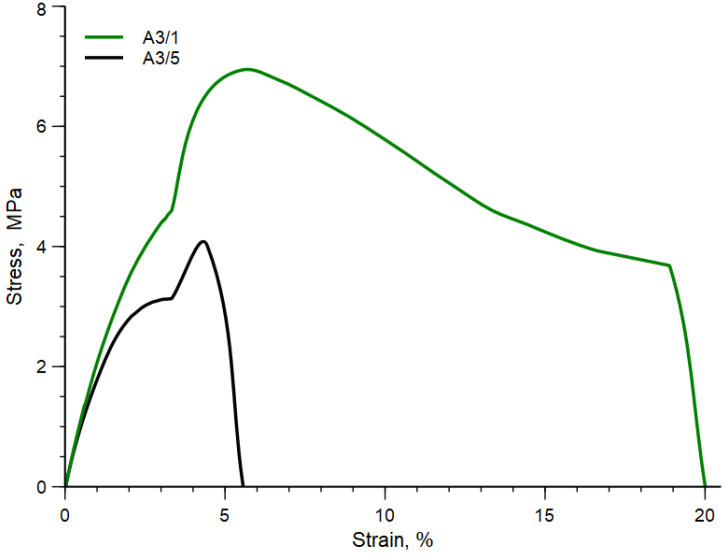
Stress-strain curve of A3/1 and A3/5 blends.

It is well evident that the blend processed 1 min shows particles of the dispersed phase having lower values of the diameter and smaller are also the voids present in the continuous phase of this specimen. The applied stress is then able to break the particles of the dispersed phases reducing their size, but also to form copolymers able to decrease the interfacial tension between matrix and dispersed phases and then improve the adhesion between matrix and dispersed phases. The numerical average diameter is about 3.5 µm and the diameter distribution decreases from 3–10 µm to 2–6 µm. The mechanical properties are reported in Table 13 for the two blends and their stress-strain curves are plotted in Figure 11. Additionally, in this Figure, the sudden change of the stress-strain curves is due to the change of the crosshead speed.

The two stress-strain curves are strongly different. Indeed, the curve relative to the A3/5 sample is the typical stress-strain curve of a fragile polymer, while the stress-strain curve of sample A3/1 shows yield stress and ductile behavior.

Elastic modulus, tensile strength and elongation at break are significantly higher for the blend processed 1 min. It is very impressive, however, that the mechanical behavior of the A3/1 blend is dramatically changed and a brittle-to-ductile transition with the presence of yield stress is observed by optimizing the processing time.

The change of morphology has implied a brittle-to-ductile transition due to the higher contact area between the matrix and dispersed particles and a better adhesion between the matrix and other phases.

This remarkable effect of time can be interpreted in two different ways. The first one is connected simply to the higher mechanical stress experienced at 1 min. The morphology of the sample is “frozen” in that obtained at the higher values of the torque. The second one can be correlated with the formation of small amounts of copolymers that, with increasing mixing time, are broken by the same mechanical stress reducing the effect of these adhesion promoters and worsening the final morphology of the blend. In addition, in this case, the ratio $\;A_{A3/1}t\;/\;A_{A3/5}t≅\;$1.83 and thus a much larger contact area with respect to the blend A3/5 and especially much larger than that of the brittle sample. This result is in accordance with the above results.

**Figure 12:**
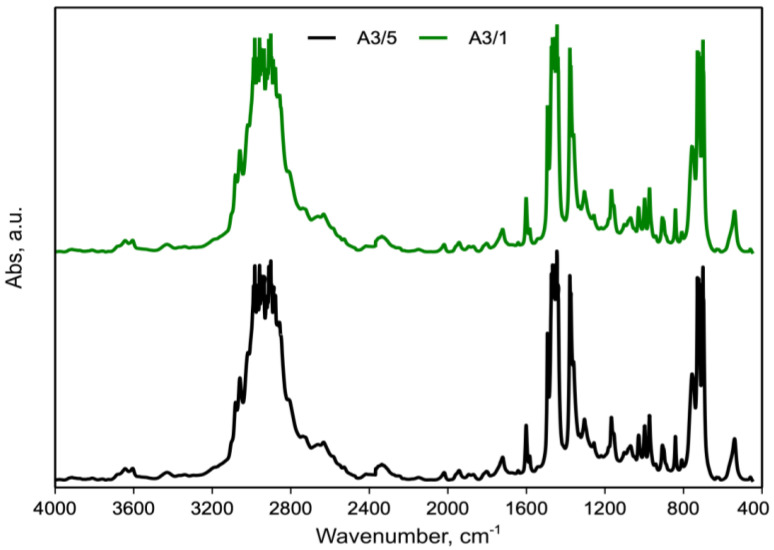
FTIR spectra of A3/1 and A3/5 blend.

Figure 12 shows the FTIR spectra of the A3/1 and A3/5 blends.

Observations similar to those reported previously can be made, as no new peaks or significant peaks shifts were observed between the A3/5 and A3/1 blend.

**Figure 13:**
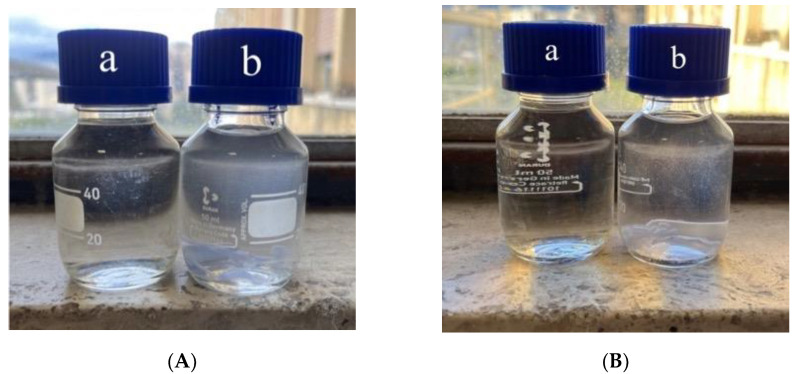
Photos of the solutions PS/PET: (**A**) PS/PET_1: (a) solvent, (b) suspensions; (**B**) PS/PET_5: (a) solvent, (b) suspension.

**Figure 14:**
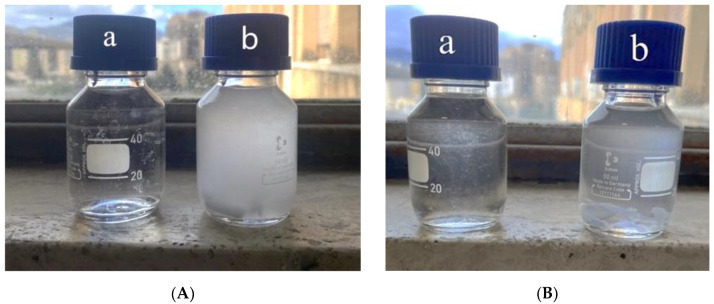
Photos of the solutions LDPE/PET: (**A**) LDPE/PET_1: (a) solvent, (b) suspensions; (**B**) LDPE/PET_5: (a) solvent, (b) suspension.

Figure 13 and Figure 14 show the photos of the solutions PS/PET and LDPE respectively for the binary blends prepared at 180 °C, 250 rpm and 1 and 5 min, while, Table 14 summarizes the turbidity values. It is evident that the two suspensions show some turbidity and this is an indication of the presence of copolymers PS-PET and LDPE-PET that are present in the form of colloids. It is also clear that the turbidity of the blends prepared for 1 min is higher than that shown of the blend mixed for 5 min. These copolymers act as compatibilizers giving a better adhesion and a better morphology.

A competition between the formation of copolymers and cleavage of the same copolymers by thermomechanical degradation can well interpret this result and the presence of a maximum for the blend prepared for 1 min as for the morphology and mechanical properties. The copolymers are formed but, at the same time, the thermomechanical stress breaks then decreasing the adhesion effect.

## 4. Conclusions

The processing of heterogeneous mixtures to produce blends with good mechanical properties is very difficult due to the incompatibility of the different components. In this case, blends with poor mechanical properties are obtained. In order to overcome this problem, it is usual to use graft or block copolymers as compatibilizers. However, when dealing with heterogeneous blends composed of more than two components, it is not always possible to have ad hoc compatibilizer, all the more so when the components come from municipal solid waste, where the chemical nature is often changed due to a degradation processes.

In this paper, the effect of temperature, rotational speed and time on the morphology, viscosity and mechanical properties of a heterogeneous blend was studied using a conventional laboratory mixer. In particular, the processing parameters were optimized to obtain high stresses able to produce particles of the dispersed phases with very low dimensions and to generate copolymers capable of acting as compatibilizers.

It has been observed that by decreasing the temperature and increasing the speed of rotation, both rheological behavior and ductility values increase thanks to a decrease in the size of the dispersed phases and to an improvement of the adhesion between the matrix and dispersed phases. The improvement of the adhesion is due to the formation of small amounts of copolymers formed between the macroradicals generated by the highly applied mechanical stress. In addition, it has been observed that by optimizing the mixing time by reducing it from 5 min to 1 min an increase in ductility of about 270% is observed with a brittle-to-ductile transition. This last effect has been interpreted considering that the copolymers formed after 1 min at the highest shear stress are broken with increasing the mixing time by the thermomechanical stress.

## Figures and Tables

**Table 1:** Main characteristic of the polymers.

Materials	Supplier	Name	Density, g/cm^3^	MFI, g/10 min	Melting Point, °C
HDPE	Versalis	Eraclene DB506	0.939	0.26	127
LDPE	Versalis	Riblene FC 30	0.922	0.25	112
PP	Lyondellbasell	Moplen RP340H	0.900	1.80	164
PET	-	-	1.380	49.0	255
PS	IneosNova	Empera 251N	1.040	2.40	-

**Table 2:** Composition of the mixture.

Materials	HDPE	LDPE	PP	PET	PS
Composition blend, %	30	30	15	15	10

**Table 3:** Temperatures used for the preparation of the blends.

Temperature, °C	180	210	240	270
	A	B	C	D

**Table 4:** Mixing speeds used for the preparation of the blends.

Speed, rpm	60	120	250
	1	2	3

**Table 5:** Blends code for all the investigated blend.

	Blends Code	
-	-	A3
-	-	B3
-	C2	C3
D1	D2	D3

**Table 6:** MFI values of all the investigated blends.

Property	A3	B3	C3	C2	D3	D2	D1
MFI, g/10 min	0.503	0.537	0.542	0.568	0.571	0.597	0.689

**Table 7:** Numerical average diameter values of A3, C2 and D1 blends.

	A3	C2	D1
Numerical average diameter, Dn, µm	6.57	8.34	12.66

**Table 8:** Total contact area value with respect to D1.

	A3	C2
Total contact area with respect to D1	1.926	1.517

**Table 9:** Torque values at 5 min for the blends A3, C2, D1.

Property	A3	C2	D1
Torque, N m	32.2 ± 1.4	24.8 ± 1.1	15.5 ± 0.9

**Table 10:** Elastic modulus, E, tensile strength, TS, and elongation at break, EB, of all the investigated blends.

Blend Code	E, MPa	TS, MPa	EB, %
A3	176 ± 19	4.2 ± 2.5	5.1 ± 1.1
B3	172 ± 23	3.5 ± 1.8	3.3 ± 0.8
C3	174 ± 18	3.8 ± 1.5	3.2 ± 1.0
C2	169 ± 23	3.5 ± 1.5	3.1 ± 0.9
D3	181 ± 27	3.9 ± 1.6	3.0 ± 0.7
D2	171 ± 24	3.5 ± 1.6	2.8 ± 0.6
D1	165 ± 22	3.0 ± 1.9	2.6 ± 0.5

**Table 11:** MFI values of A3/1 and A3/5 blends.

Property	A3/1	A3/5
MFI, g/10 min	0.425	0.503

**Table 12:** Numerical average diameter values of A3/1 and A3/5 blends.

	A3/1	A3/5
Numerical average diameter, Dn, um	3.58	6.57

**Table 13:** Tensile properties of A3/1 and A3/5 blends.

Blend Code	E, MPa	TS, MPa	EB, %
A3/1	212 ± 13	7.6 ± 2.5	19.1 ± 4.1
A3/5	176 ± 19	4.2 ± 2.5	5.1 ± 1.5

**Table 14:** Degree of turbidity of suspensions.

Turbidity, FTU	PS/PET_1	PS/PET_5	LDPE/PET_1	LDPE/PET_5
Suspensions	9.21 ± 1.1	3.16 ± 0.8	36.9 ± 7.8	10.5 ± 2.6

## Data Availability

The date presented in this work are available on request from the corresponding author.
